# Oxidative Stress in Health and Disease

**DOI:** 10.3390/biomedicines11112925

**Published:** 2023-10-29

**Authors:** V. Prakash Reddy

**Affiliations:** Department of Chemistry, Missouri University of Science and Technology, Rolla, MO 65409, USA; preddy@mst.edu; Tel.: +1-(573)-341-4768

**Keywords:** oxidative stress, Alzheimer’s disease, diabetes, reactive oxygen species, reactive nitrogen species, 4-hydroxy-*trans*-2-nonenal (HNE), lipid peroxidation, nanozymes, receptors for advanced glycation end products (RAGE)

## Abstract

Oxidative stress, resulting from the excessive intracellular accumulation of reactive oxygen species (ROS), reactive nitrogen species (RNS), and other free radical species, contributes to the onset and progression of various diseases, including diabetes, obesity, diabetic nephropathy, diabetic neuropathy, and neurological diseases, such as Alzheimer’s disease (AD), amyotrophic lateral sclerosis (ALS), and Parkinson’s disease (PD). Oxidative stress is also implicated in cardiovascular disease and cancer. Exacerbated oxidative stress leads to the accelerated formation of advanced glycation end products (AGEs), a complex mixture of crosslinked proteins and protein modifications. Relatively high levels of AGEs are generated in diabetes, obesity, AD, and other I neurological diseases. AGEs such as N^e^-carboxymethyllysine (CML) serve as markers for disease progression. AGEs, through interaction with receptors for advanced glycation end products (RAGE), initiate a cascade of deleterious signaling events to form inflammatory cytokines, and thereby further exacerbate oxidative stress in a vicious cycle. AGE inhibitors, AGE breakers, and RAGE inhibitors are therefore potential therapeutic agents for multiple diseases, including diabetes and AD. The complexity of the AGEs and the lack of well-established mechanisms for AGE formation are largely responsible for the lack of effective therapeutics targeting oxidative stress and AGE-related diseases. This review addresses the role of oxidative stress in the pathogenesis of AGE-related chronic diseases, including diabetes and neurological disorders, and recent progress in the development of therapeutics based on antioxidants, AGE breakers and RAGE inhibitors. Furthermore, this review outlines therapeutic strategies based on single-atom nanozymes that attenuate oxidative stress through the sequestering of reactive oxygen species (ROS) and reactive nitrogen species (RNS).

## 1. Introduction

Oxidative stress is a causative factor for the onset of diabetes, obesity, and diabetes-induced microvascular diseases, including diabetic retinopathy end-stage renal disease, atherosclerosis, and cardiovascular diseases [1,2,3,4]. Oxidative stress results from the excessive intracellular accumulation of reactive free radicals, such as reactive oxygen species (ROS) and reactive nitrogen species (RNS). Whereas ROS and RNS are an integral part of normal cellular function and host defense system against the invading bacteria, their excessive amounts contribute to the onset and exacerbation of various pathologies, including neurological diseases and diabetes [5,6,7,8]. 

Cellular antioxidants, such as glutathione, and antioxidant enzymes, such as superoxide dismutase (SOD), catalase (XAT), glutathione peroxidase (GPx), sequestrate ROS and RNS to maintain an optimal balance of the cellular redox status [9,10]. The imbalance between the formation and destruction of ROS and RNS contributes to the excessive accumulation of these reactive free radicals. Although ROS and RNS act as signaling molecules under physiological concentrations, under oxidative conditions, excessive amounts of ROS and RNS exert cellular damage through their deleterious reactions with proteins, lipids, and DNA, thereby leading to the pathogenesis of various oxidative stress-related diseases, including diabetes, obesity, and neurological diseases, such as Alzheimer’s disease (AD) and Parkinson’s disease (PD) [11,12,13]. 

Oxidative stress also contributes to the formation of advanced glycation end products (AGEs), and thereby the deleterious structural modifications of proteins and nucleic acids. AGEs are formed through nonenzymatic reactions of protein amino groups with the carbonyl groups of the reducing sugars, followed by further protein modifications involving glycoxidation reactions, and thus the oxidative stress plays a major role in the formation of AGEs and the onset of AGE-related diseases, including AD, diabetes, atherosclerosis, and amyotrophic lateral sclerosis (ALS) (vide infra) [14,15]. Relatively high levels of AGEs are ubiquitously found in diabetes, obesity, AD, and other neurological diseases, and AGEs are mainly localized in the amyloid beta (Aβ) plaques and neurofibrillary tangles (NFT) in cases of AD [16,17]. The levels of AGEs are correlated with the extent of oxidative stress and disease progression in diabetes, obesity, and Alzheimer’s disease (AD). 

Diabetes is also one of the major causative factors for the onset of Alzheimer’s disease (AD), and they have common biomarkers, including elevated amounts of AGEs and other oxidative stress markers, such as 4-hydroxy-*trans*-2-nonenal (HNE), a lipid peroxidation product [18,19,20,21]. Because of the common biomarkers of AD and type 2 diabetes, AD is sometimes referred to as diabetes 3 [22,23,24,25,26,27]. 

Excessive amounts of AGEs and their binding to receptors for AGEs (RAGE) induce signaling cascades that can further exacerbate oxidative stress, in a vicious cycle. The interactions of AGEs with RAGE (AGE–RAGE interactions) are also involved in the onset of pancreatic cancers under hyperglycemic conditions, especially in cases of diabetes and obesity [28]. AGE–RAGE interactions and the resulting oxidative stress are causative factors in the onset of diabetic kidney disease (vide infra). 

Oxidative stress also generates a complex mixture of lipid peroxidation products, some of which, such as malondialdehyde (MDA) and 4-hydroxy-*trans*-2-nonenal (HNE) are primarily responsible for DNA modifications and the resulting carcinogenesis (vide infra) [29]. There is a renewed interest in developing therapeutics targeted at the sequestration of lipid peroxidation products and antioxidants that would attenuate lipid peroxidation [30].

There is also an emerging interest in developing RAGE inhibitors as potential therapeutics for cancers and neurological diseases, as well as other AGE-related diseases. There have been no FDA-approved RAGE inhibitor-based therapeutics to date because RAGE is a multi-ligand binding receptor, and achieving selectivity for RAGE binding is still a challenge. However, there are potentially useful therapeutics that are currently in clinical trials for treating cancers and neurological diseases (vide infra) [14,17,31,32,33,34].

Nanozymes that are engineered to selectively enter neuronal cells are effective in attenuating the oxidative stress, and this area has attracted emerging interest toward developing effective therapeutics for treating various neurological diseases. One recent trend in this area is to embed single-atom-based nanozymes on the surface of near-infrared probes for the purposes of imaging the sites of neuroinflammation. Nanozyme materials made from redox-active metals, such as Mn, Co, Zn and Pt, attenuate the neuroinflammation, annihilate tumor cells, and mediate diabetic wound healing by sequestering ROS or acting as antibacterial agents [5,35,36,37,38,39]. These nanozymes can also be engineered such that they permeate through the blood–brain barrier (BBB) and show high selectivity for entry into the neuronal cells when targeting neurological diseases, such as traumatic brain injury (TBI) (vide infra) [39,40].

## 2. Reactive Oxygen Species (ROS)

Reactive oxygen species (ROS) are formed physiologically during the mitochondrial respiratory cycle, as well as during the cellular metabolism and nonenzymatically through transition-metal-ion-catalyzed redox reactions (Fenton reaction). The mitochondria are a major source of ROS formation, and mitochondrial antioxidant enzymes—Mn superoxide dismutase (MnSOD), catalase, and ascorbate oxidase—control the levels of mitochondrial ROS in order to maintain the optimal balance of ROS necessary for normal physiological activity. ROS and the ensuing oxidative stress are involved in the onset of various diseases, including diabetes, cardiovascular disease, diabetic neuropathy, and diabetic nephropathy. Hyperglycemia, a primary cause of diabetes, activates various signaling pathways, leading to the overexpression of ROS and oxidative stress. Increased oxidative stress leads to endothelial dysfunction and atherosclerosis in cases of diabetes [41]. In transgenic animal models overexpressing MnSOD, it was shown that the attenuation of mitochondrial ROS in endothelial cells improves coronary angiogenesis and cardiac function in non-reperfused mitochondrial infarction [42].

TBI involves extensive impairment of the BBB accompanied by the excessive production of ROS, such as superoxide radical anions (O_2_·^−^), hydroxyl radicals (·OH), and hydrogen peroxide (H_2_O_2_). ROS-induced neuronal cell damage leads to long-term effects on health and leads to the onset of AD in some cases. 

**Figure 1:**
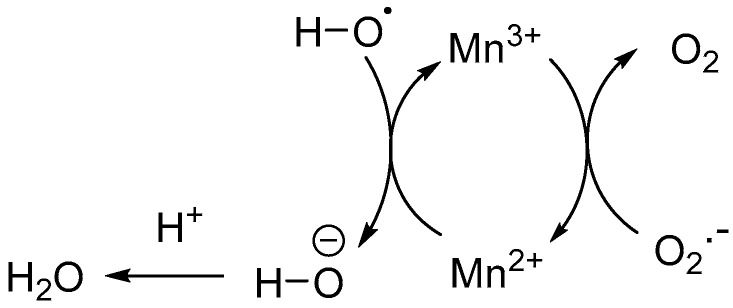
Single-atom Mn catalyst embedded in a Ag_2_Te near-infrared probe for the sequestration of intracellular ROS; the redox-active Mn^2+/3+^, through a single-electron transfer to a hydroxyl radical and superoxide radical anion, forms the non-harmful hydroxide anion and dioxygen, respectively.

Oxidative stress disrupts the BBB, thereby further exacerbating neuronal damage in AD and TBI cases [43]. Sequestration of ROS by a single-atom Mn catalyst alleviates neuroinflammation and promotes reconstruction of the BBB, accompanied by the recovery of neurological function [40]. This single-atom Mn catalyst was embedded in a near-infrared-II (1500–1700 nm) silver telluride (Ag_2_Te) quantum dot as an imaging probe for monitoring the neuroinflammation induced by ROS. In this nanozyme-type catalytic system, the redox-active Mn transforms the superoxide radical anions (O_2_·^−^) to dioxygen (O_2_), and the hydroxyl radicals (·OH) to hydroxyl anions (^−^OH), which are subsequently transformed into H_2_O by abstracting a proton from the neighboring acidic sites (Figure 1). A similar approach using Mn doped onto the near IR-II (1500–1700 nm) active PbS/CdS quantum dot (QD) imaging agent inhibits the release of pro-inflammatory factors and sequestrates the excessive ROS in TBI brains, thereby affording neuroprotective effects [39]. Therapeutic candidates using single-atom based catalytic nanozymes are currently in development for the treatment of neurological diseases, including AD and TBI [38]. Furthermore, nanozymes can be designed such that they cross the BBB and eliminate the misfolded proteins, and therefore may serve as effective therapeutics, in particular for the neurological diseases [37].

Single-atom catalysts consisting of Pt/CeO_2_, in addition to breaking down the ROS, contribute to the blocking of the source of ROS generation in the mitochondria. Thus, these single-atom catalysts achieve the self-clearance of dysfunctional mitochondria by interfering with the α-glycerophosphate shuttle pathway and malate-aspartate shuttle pathway, thereby attenuating ROS re-generation [44]. Naturally occurring antioxidants, such as lycopene, are potentially useful for the sequestration of ROS. However, the use of naturally occurring antioxidants in the treatment of Parkinson’s disease (PD) is hampered owing to the technical challenges involved in their incorporation into the PD neuronal cells. In this context, biocompatible lycopene-based anti-ROS nanodots, when engineered to target the neuronal mitochondria, induce the efflux of the pathogenic α-synuclein and aid in the survival of the dopaminergic neurons in PD [45].

**Figure 2:**
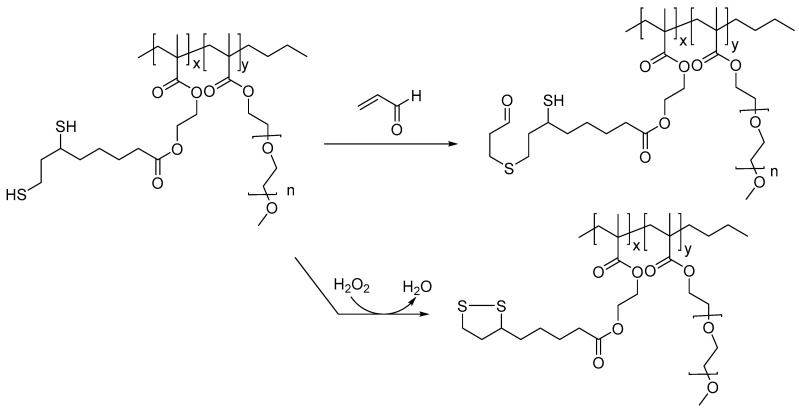
Lipoic-acid-based polymer and proposed mechanism for the sequestration of ROS and lipid peroxidation products; such multi-target-based therapeutics candidates, when conjugated to imaging agents, could serve as theranostics for treating TBI.

A lipoic acid-derived methacrylate co-polymer was shown to scavenge lipid peroxidation products, such as acrolein, and H_2_O_2_. Such compounds, when used as therapeutic candidates, may provide protective effects in TBI and other neurodegenerative diseases, as multiple lipid peroxidation products and excessive ROS are generated in cases of TBI and AD (Figure 2) [30]. Presumably, toxic levels of H_2_O_2_ are quenched through oxidation of the dithiol moiety in the polymer to the disulfide. These polymers were also integrated with Gd-DOTA-based MRI-imaging agents toward monitoring their potential therapeutic effects. 

**Figure 3:**
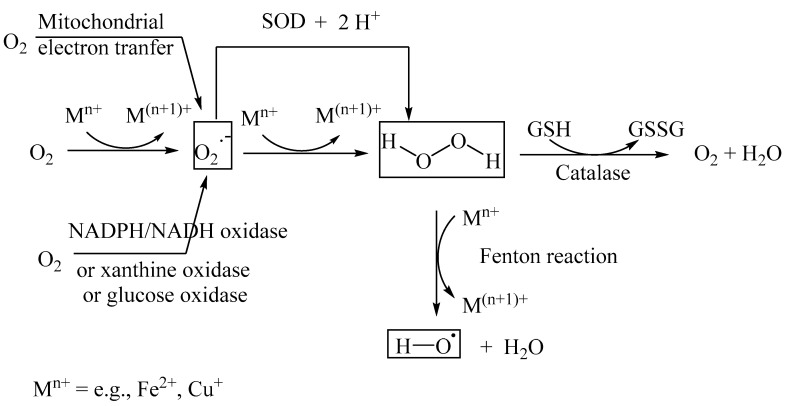
Intracellular formation of ROS (superoxide radical anion and hydroxyl radical); a superoxide radical anion (O_2_·^−^) is formed from molecular oxygen either through an Fe(II)-catalyzed Fenton reaction or through an enzyme-catalyzed single electron transfer from NADPH (or NADH). The Fenton reaction of O_2_·^−^ forms the highly reactive hydroxyl radial (HO·) through the intermediate formation of H_2_O_2_, which is also classified as a ROS.

Superoxide radical anions (O_2_·^−^) are generated either enzymatically, catalyzed by nicotinamide adenine dinucleotide (NADH or NAD-phosphate, NADPH) oxidase, glucose oxidase, or xanthine oxidase [46,47,48], or through the non-enzymatic Fenton reaction, catalyzed by metal ions, such as Fe(II) and Cu(I) [2]. The superoxide radical anion, O_2_·^−^, is relatively less reactive and thus, by itself, is not a major contributor to the initiation of oxidative stress. However, the superoxide radial anion forms a highly reactive hydroxyl radical species (HO·) upon further reductive transformation via SOD into H_2_O_2_, followed by a metal-ion-catalyzed Fenton reaction. The hydroxyl radical is substantially more reactive than the superoxide radical anion and reacts with proteins and nucleic acids to form various protein and nucleic acid aggregates, which are implicated in a broad variety of pathologies, including diabetes, obesity, and neurological diseases. Catalase transforms the relatively toxic hydrogen peroxide into dioxygen and H_2_O (Figure 3). Other antioxidant enzymes that attenuate ROS include glutathione peroxidase, CuZn-SOD, and Mn-SOD. Excessive amounts of ROS, such as the hydroxyl radical, accumulate and initiate oxidative stress when there is an imbalance in the formation and sequestration of ROS. In cases of diabetes, the pancreas has relatively low levels of antioxidant enzymes and a high glucose concentration, and therefore is prone to ROS-induced damage of the insulin-producing B cells [49]. 

## 3. Lipid Peroxidation

**Figure 4:**
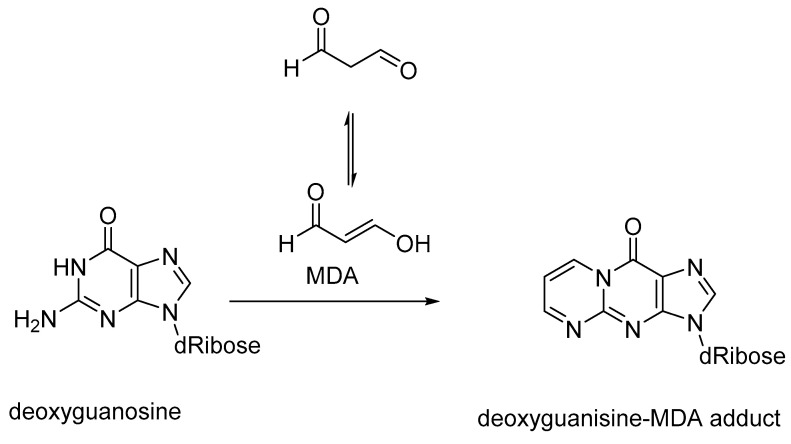
Formation of an MDA adduct from deoxyguanosine; MDA is used as a marker of oxidative stress and lipid peroxidation.

The reaction of the hydroxyl radicals with lipids forms lipid hydroperoxides through chain-propagating events. HNE, formed from the lipid peroxidation of linoleic acid, exerts cytotoxicity through binding to nucleic acid bases and proteins. The nucleic acid adducts of HNE contribute to mutagenesis and carcinogenicity, and the protein modifications result in the loss of protein function or enzyme deactivation. Lipid peroxidation of polyunsaturated fatty acids generates MDA, which forms nucleic acid-base adducts. In solution, MDA exists in the enol tautomeric form. Deoxy-guanosine forms adducts with MDA on the pyrimidine ring to give pyrimido[1,2a]purin-10(3H)-one as the major product (Figure 4). MDA also forms adducts with deoxyadenosine and deoxycytidine, which in the absence of intracellular DNA repair mechanisms would result in mutagenicity and carcinogenicity [50]. MDA levels are elevated in type 2 diabetes cases with coronary artery disease, and MDA is also used as a marker of oxidative stress and lipid peroxidation [51].

Some of the lipid peroxidation products, such as HNE, are abundantly found in cases of diabetes and Alzheimer’s disease, meaning that HNE is used as a biomarker for the progression of disease [52]. HNE reacts with proteins and nucleic acids to produce covalent adducts, and these protein and nucleic acid modifications can lead to the onset of various pathologies, including AD and diabetes. Hyperglycemia elevates the levels of HNE in diabetic patients. HNE correlates with the production of Aβ peptide aggregates and may trigger the onset of AD in cases of hyperglycemia [53]. HNE acts as a cell-signaling molecule and thereby plays a role in the onset of hepatocellular carcinoma, pancreatic cancer, and colorectal cancer in diabetic patients [54]. HNE is also involved in the onset and progression of pulmonary fibrosis, a disease in which there is an excessive accumulation of extracellular matrix in the lung tissues [55]. HNE levels are elevated in osteoarthritis cases, and it was hypothesized that HNE induces transcriptional and posttranslational modifications of collagen II and matrix metalloproteases 13 in chondrocytes, thereby affecting collagen homeostasis and collagen degradation [56,57].

**Figure 5:**
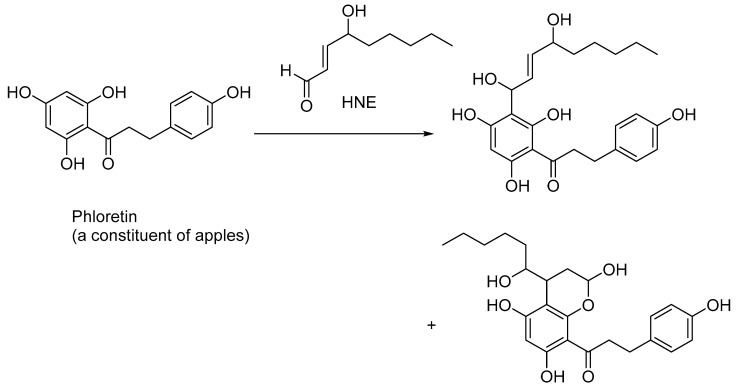
Sequestration of HNE by phloretin, a polyphenolic antioxidant found in apples; other naturally occurring polyphenolic compounds, such as resveratrol, also sequester HNE and other lipid peroxidation products, such as acrolein and MDA.

Naturally occurring polyphenols sequester HNE and, to some extent, attenuate the toxicity effects of HNE. Thus, phloretin, a polyphenolic constituent of apples, undergoes the Friedel–Crafts-type reaction with the activated aromatic ring to produce benzylic alcohol along with other hemiacetal compounds, as shown in in vitro experiments (Figure 5) [58]. A dose-dependent trapping of HNE in phloretin-fed mice was also demonstrated [58].

**Figure 6:**
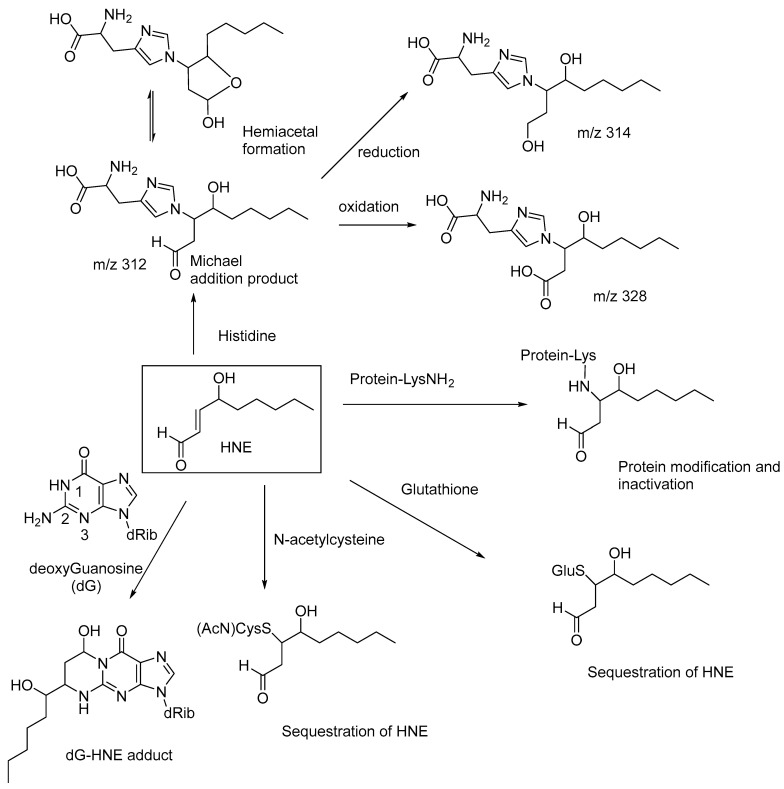
Cellular toxicity of HNE through protein and DNA modifications and sequestration of HNE by glutathione and N-acetylcysteine; HNE and its DNA-based adducts are also used as biomarkers for monitoring the progression of human cancers.

N-acetylcysteine, acting as an antioxidant, may also help to decrease the levels of HNE and potentiate the antiarthritic effect of epalrestat, an aldose reductase inhibitor, when used as a combination drug [59]. Similarly, glutathione also undergoes Michael addition to HNE to form a nontoxic adduct, which is reduced in vivo to the corresponding alcohol [60,61]. Michael addition of HNE to the N^2^-amino group of deoxyguanosine, followed by cyclic hemiaminal formation, forms the dG-HNE adduct (Figure 6) [62]. These observations indicate that the cellular toxicity of HNE is due to the conjugate addition (Michael addition) of the amino groups of proteins and nucleic acids (i.e., the nucleophilic addition of the amino groups at the β-carbon of HNE), and thereby affecting their normal physiological functions and nucleic acid replication and transcription. HNE also forms Michael adducts with proteins through reactions with the histidine sidechains, as evidenced in high-performance liquid chromatography-tandem mass spectroscopy (LC-MS/MS) studies. The Michael addition product of histidine reversibly undergoes cyclization to form the corresponding hemiacetal (*m*/*z* 312), and the adduct is enzymatically reduced to the corresponding alcohol (*m*/*z* 314) and oxidized to the corresponding carboxylic acid (*m*/*z* 328; Figure 6) [63]. These compounds serve as biomarkers for lipid-peroxidation-derived carbonyl stress.

HNE forms adducts with all four nucleic acid bases and alters their transcriptional properties, resulting in cancerous mutations [64]. These nucleic acid–HNE adducts, together with the oxidative stress-mediated factors, contribute to the onset of liver cancer. The DNA-base adducts of HNE may be used as biomarkers for lipid-peroxidation-mediated DNA damage in human cancers.

The antioxidants N-acetylcysteine (NAC) and glutathione have relatively high reactivities with Michael additions on HNE as compared to those of proteins and nucleic acids and effectively sequester the HNE, thereby attenuating the toxicity of HNE (Figure 6). ROS, generated through cigarette smoking, upregulate angiotensin-converting enzyme 2 (ACE2) in the alveolar macrophages (AMs) and thereby increase the susceptibility of AMs to SARS-CoV-2 infection. NAC decreases ACE2 expression by suppressing intracellular ROS, and thus NAC and other ROS-sequestrating antioxidants may show a preventive effect for the pulmonary complications of COVID-19 [65].

## 4. ROS and DNA Damage

**Figure 7:**
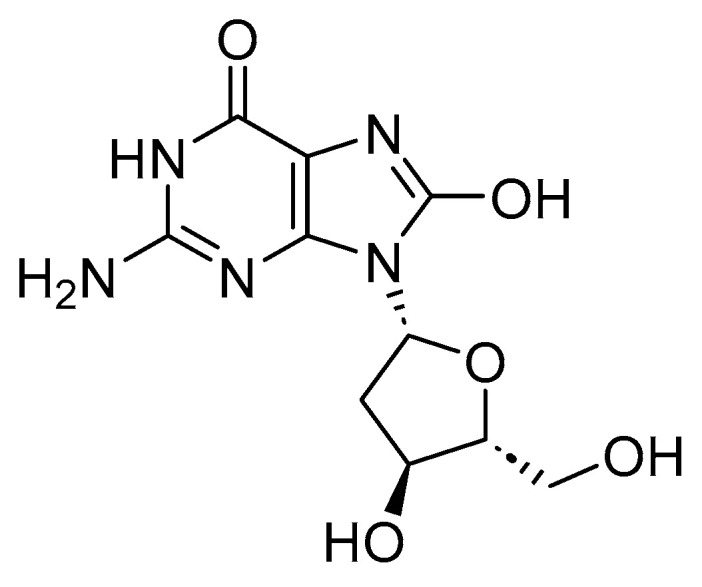
Structure of 8-hydroxy-2′deoxyguanosine (8-oxo-dG); 8-oxo-dG serves as a marker for oxidative stress and DNA damage.

ROS oxidizes 2′-deoxy-guanosine to 8-hydroxy-2′deoxyguanosine (8-oxo-dG; Figure 7), thereby causing site-specific DNA damage. 8-oxo-dG is used as a marker for oxidative stress and DNA damage. It was shown that myricetin, a constituent of tea and berries, may cause DNA damage induced by H_2_O_2_, in the presence of Cu(I), presumably through the in situ formation of Cu(I)-hydroperoxide [66]. Cancer drugs, such as diosgenin, promote DNA damage in cancerous cells, mediated by ROS. Co-treatment using diosgenin and cisplatin resulted in increased DNA damage, increased levels of ROS, and decreased cellular antioxidant enzymes, thereby inducing apoptosis in the tumor cells [67].

## 5. Reactive Nitrogen Species (RNS)

**Figure 8:**
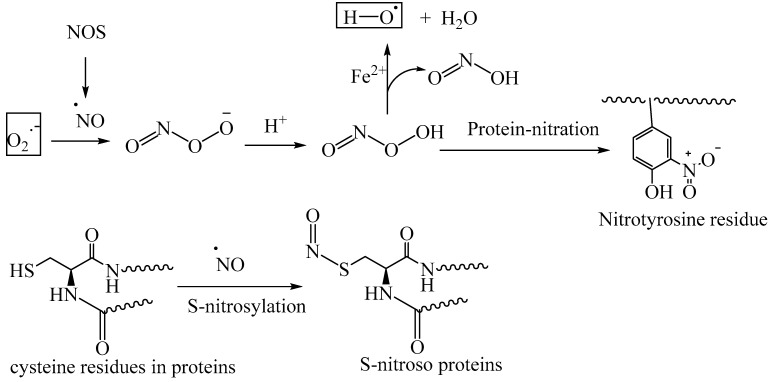
Reactive nitrogen species and their effect on protein modifications; nitric oxide exists as a radical species and acts as a signaling molecule in various physiological processes.

Reactive nitrogen species (RNS) include the free radical nitric oxide (NO) and peroxynitrous acid (ONOOH). Nitric oxide synthase (NOS) catalyzes the oxidative transformation of L-arginine into NO, using NADPH as a cofactor. NO serves as a signaling molecule under physiological concentration. However, the aberrant formation of excessive NO through overactivation of NOS leads to oxidative stress. The reaction of NO with superoxide forms peroxynitrous acid. Peroxynitrous acid is involved in the nitration of the tyrosine residues of the proteins, thereby altering the protein function (nitrative stress) (Figure 8) [68].

Nitric oxide is involved in the nitrosylation of the thiol residues of proteins, thereby exerting nitrosative stress. Under normal physiological conditions, S-nitrosylated proteins mediate redox signaling and control the cellular metabolism. However, under oxidative stress conditions (i.e., excessive production of NO), nitrosative stress contributes to the pathophysiology of various diseases, including AD, Parkinson’s disease, and Huntington’s disease [69,70,71]. In the case of AD and Parkinson’s disease, there is a correlation between the S-nitrosylation of the redox enzyme protein disulfide isomerase (PDI) and endoplasmic stress [71]. Peroxynitrous acid also generates the highly reactive hydroxyl radical through the metal-ion-catalyzed Fenton reaction, further increasing oxidative stress (Figure 8).

Nitrotyrosine was found in cases of AD, amyotrophic lateral sclerosis (ALS), and multiple sclerosis, and is used as a marker of these diseases, although there is no clear evidence of whether these markers are the cause or epiphenomena of these diseases [72]. Nitrosative and nitrative stress are contributing factors to the neurodegeneration observed in multiple sclerosis [73]. Therapeutics targeted at the modulation of NOS have not yet been approved for clinical use, although selective NOS inhibitors may be developed for treating multiple diseases, such as AD and diabetes [74].

## 6. Oxidative Stress and Advanced Glycation End Products (AGEs)

AGEs are abundantly formed in a multitude of diseases, including diabetes, and neurological disorders, such as AD and Parkinson’s disease. AGEs are formed through nonenzymatic reactions of the reducing sugars with the primary amino groups of proteins, followed by a series of oxidative transformations. These oxidative transformations, called glycoxidations, are exacerbated under oxidative stress conditions, i.e., when reactive oxygen species (ROS) and RNS are formed in excessive amounts through aberrant cell metabolism [15,75,76]. The intermediary 1,2-dicarbonyl products of the Maillard reaction, such as methylglyoxal, glyoxal, and 2-glucosone, are highly reactive with the protein amino groups to form protein crosslinks, thereby inactivating the enzymes. The excessive accumulation of these 1,2-dicarbonyl compounds (α-dicarbonyl compounds), also referred to as carbonyl stress, contributes to the formation of AGEs and protein crosslinks. The accumulation of AGEs, and in turn AGE–RAGE interactions, exacerbates oxidative stress, carbonyl stress, and other pathogenic factors, including the imbalance of the gut microbiota. Cereal polyphenolic compounds, through their antioxidative effects, provide a means of nonpharmacological intervention in attenuating oxidative stress and carbonyl stress, and thereby represent a preventive approach for the treatment of diabetes and neurological diseases [77].

**Figure 9:**
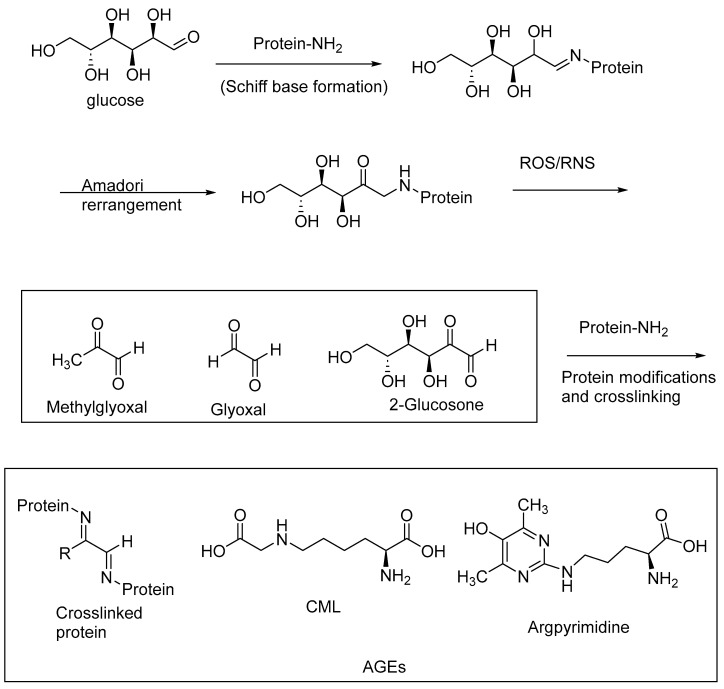
Schematic representation of AGE formation through nonenzymatic glycation and glycoxidation; oxidative stress contributes to the glycoxidation reactions of the Schiff base adducts of reducing sugars and protein amino groups, forming AGE-crosslinked proteins and AGE-protein modifications.

AGEs may be formed as intramolecular or intermolecular protein crosslinks (e.g., pentosidine, a lysine and arginine crosslink) or protein modifications involving lysine or arginine side chains (e.g., argpyrimidine, N^e^-carboxymethyllysine (CML)) (Figure 9). AGE levels are correlated with an increasing severity of diabetes, diabetic neuropathy, diabetic nephropathy, and neurological diseases, including AD and PD.

AGEs accelerate diabetes-related atherosclerosis by activating RAGE-NF-kB signaling, thereby promoting low-density lipoprotein (LDL) transcytosis in endothelial cells [78]. AGE inhibitors, such as pyidoxamine, sequester the intermediary AGE precursor methyl glyoxal and other 1,2-dicarbonyl compounds, and thereby prevent atherosclerosis formation [79]. In vitro studies have shown that AGEs enhance the activation of NADPH oxidase and thereby ROS generation in the endothelial cells. Losartan, ramipril, resveratrol, and N-acetylcysteimiine (NAC) attenuated AGE-induced endothelial dysfunction, presumably through their antioxidant effects and ROS sequestration [80].

AGEs are also formed during the high-temperature processing of foods. These dietary AGEs, when ingested, may also contribute to disease onset and the progression of non-alcoholic fatty liver disease (NAFLD) [81], pediatric obesity [82], cancer [83], dementia [84], and diabetes [85].

## 7. AGE Inhibitors as Therapeutic Targets

**Figure 10:**
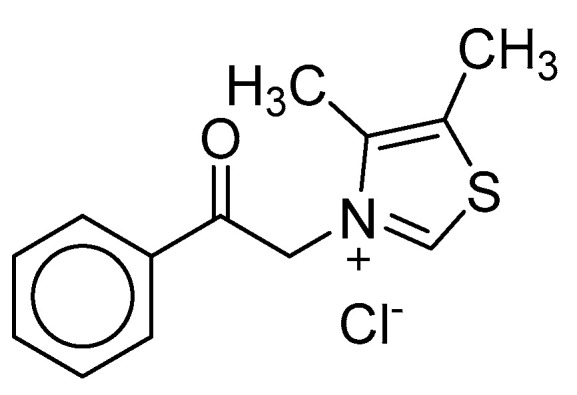
Structure of alagebrium (ALT-711), an AGE-crosslink breaker; clinical trials of this drug candidate were terminated due to its adverse effects.

Thiazolium-based compounds, such as ALT-711 (Alagebrium; Alteon, Inc., San Jose CA, USA), break pre-formed AGE-protein cross links. Such AGE-crosslink breakers are potentially useful as therapeutics for treating AGE-related diseases (Figure 10). Clinical trials of ALT-711 showed that it is effective in attenuating systolic blood pressure and has a positive outcome in cases of diastolic heart failure [86,87].

Aminoguanidine (pimagedine), in vitro, showed anti-glycating and AGE-inhibitory effects. However, in clinical trials, this compound showed little positive outcome, and clinical trials were terminated because of the unfavorable risk-to-benefit ratio [88,89].

Although the concept of AGE breakers and AGE inhibitors in attenuating AGEs and AGE-related diseases is promising for the future development of therapeutics for diabetes and other AGE-related diseases, currently there are no FDA-approved candidates based on AGE inhibitors or breakers. This may be attributed to the complexity of the structures of AGEs and the poorly established mechanistic pathways of their formation to date. Targeting oxidative stress resulting from AGE–RAGE interactions, as described below, may be an alternative approach for developing therapeutics based on the AGE-induced oxidative stress.

## 8. AGE–RAGE Interactions and Oxidative Stress

AGEs, through interactions with receptors for AGEs (RAGE), initiate further oxidative stress through a series of signaling cascades [14,90]. AGE–RAGE interactions lead to the activation of NADPH oxidase, and thereby increased ROS production and lipid peroxidation. AGE–RAGE interactions also result in the activation of nuclear factor kappa beta (NFkB), and thereby lead to gene activation for the upregulation of proinflammatory cytokines, including interleukin-1β (IL-1β), interleukin-6 (IL-6), and tumor necrosis factor alpha (TNF-α) [91].

AGE–RAGE interactions lead to the elevated production of matrix metalloproteinases, which are involved in the progression of aortic aneurisms. The increased oxidative stress resulting from AGE–RAGE interactions thus plays a role in aortic aneurysms [92]. In accordance with this correlation between AGE–RAGE interactions and aortic aneurysms, AGE–RAGE stress, cytokines and matrix metalloproteinases are elevated in cases of aortic aneurisms.

Increased oxidative stress leads to AGE accumulation, which in turn can exacerbate oxidative stress through their binding to RAGE. High AGE levels are correlated with RAGE expression. RAGEs also exacerbate oxidative stress through binding to High Mobility Group Box-1 (HMGB-1) proteins. The AGE-RAGE/HMGB-1 signaling pathway is involved in the onset of type 2 diabetic cardiomyopathy. About two-thirds of type 2 diabetic patients develop diabetic cardiomyopathy. Artemisinin, an antimalarial drug, was shown to attenuate oxidative stress induced by the AGE-RAGE/HMGB-1 signaling pathway and improve diabetic cardiomyopathy [93].

Dietary polyphenols scavenge ROS and thereby attenuate the formation of the reactive α-dicarbonyl compounds, whose high reactivity with the protein amino groups leads to the formation of intramolecular and intermolecular protein crosslinks and AGEs. Polyphenolic antioxidants also regulate the AGE–RAGE axis and the microbiota–gut–brain axis, thereby preventing neurodegenerative diseases, including AD, ALS, and PD [94,95].

## 9. RAGE Inhibitors as Therapeutic Candidates

RAGE inhibitors bind to RAGE and thereby attenuate the binding of RAGE to AGEs and other ligands, including amyloid beta peptide (Aβ). RAGE inhibitors can attenuate AGE–RAGE interactions, and thereby the resulting oxidative stress effects will be substantially diminished. RAGE inhibitors, therefore, have potential therapeutic effects on multiple AGE-related diseases, including neurodegenerative diseases and diabetes.

**Figure 11:**
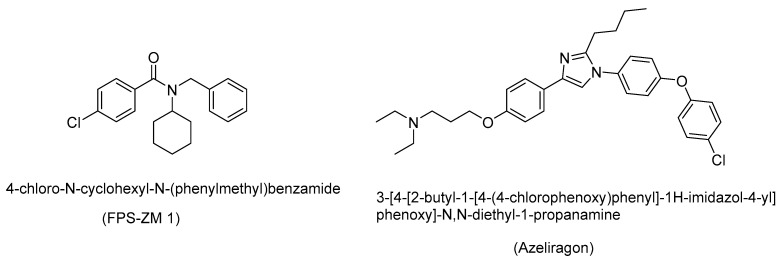
Structure of the RAGE inhibitors FPS-ZM 1 and azeliragon; clinical trials of such RAGE inhibitors are ongoing to treat various diseases, including cancers.

A RAGE inhibitor, FPS-ZM 1 (4-chloro-N-cyclohexyl-N-(phenylmethyl)benzamide), attenuates AGE-induced neuroinflammation and oxidative stress, as shown in vitro in the primary microglia of rats (Figure 11) [96]. FPS-ZM 1 attenuated AGE-stimulated NADPH oxidase and ROS expression, and thereby exhibited neuroprotective effects. FPS-ZM 1 binds to the V-domain of RAGE, and this RAGE-inhibitory effect resulted in the suppression of the influx of circulating Aβ_1-40_ and Aβ_1-42_ into the brain in a mouse model of AD. This blockade of RAGE reduced the Aβ_1-42_ and Aβ_1-40_ levels in the brain and improved the cognitive performance in the mouse model [17].

The RAGE inhibitor azeliragon (IUPAC name: 3-[4-[2-butyl-1-[4-(4-chlorophenoxy)phenyl]-1H-imidazol-4-yl]phenoxy]-N,N-diethyl-1-propanamine; also called TTP488) suppresses metastasis in triple-negative breast cancer, and displayed a favorable safety profile in Phase II clinical trials. This RAGE-inhibitor strategy may lead to small-molecule-based therapeutical candidates for treating various oxidative-stress-mediated diseases, including AD [31,97].

RAGE has several ligands, including Aβ, which is abundantly generated in AD. Inhibiting RAGE with RAGE inhibitor compounds prevents RAGE-mediated signaling for the expression of inflammatory cytokines, and therefore RAGE inhibitors may prove to be clinically useful compounds for treating AD and other AGE-related diseases, including diabetes and cancer. In animal models, and in clinical trials, azeliragon has been shown to attenuate multiple pathological pathways in AD [97,98,99].

## 10. Conclusions and Outlook

Oxidative stress is the driving force for the onset and progression of multiple diseases, including diabetes, obesity, and AD. ROS and RNS play a major role in the structural modification of proteins, nucleic acids, and lipids. The latter structural modifications of proteins and nucleic acids contribute to the pathological onset of diabetes, cancer, AD, and cardiovascular diseases. Oxidative stress also leads to the excessive formation of AGEs, and thereby the over-expression of RAGE. Paradoxically, the binding of AGEs to RAGE further exacerbates oxidative stress through a series of signaling cascades, and this AGE–RAGE interaction and the ensuing signaling cascade for the release of inflammatory cytokines and nuclear transcription factors are leading causes for the onset of pancreatic cancers in cases of diabetes and obesity.

Current therapeutic approaches involving AGE inhibitors and AGE breaker compounds, such as aminoguanidine and alagebrium (ALT-711), have displayed limited success in clinical trials. There is an increasing effort to develop RAGE inhibitors as therapeutics for AGE-related diseases, including AD and diabetes. Small-molecule-based RAGE inhibitors, such as FPS-ZM 1 and azeliragon, are currently in various stages of clinical trials for treating AD and cancer. As shown in vivo in AD models of mice, FPS-ZM 1 attenuates the influx of circulating Aβ_1-40_ and Aβ_1-42_ into the brain and improves cognitive performance. Therapeutics based on small-molecule-based antioxidants and nanozymes that can sequestrate ROS and RNS have potential impacts in drug discovery for diabetes and neurological diseases.

AGE inhibitors and breakers and RAGE inhibitors have potential impacts in treating oxidative-stress-mediated diseases, including diabetes and AD. There is a renewed interest in developing small-molecule-based RAGE inhibitors for treating cancers and neurological diseases, encouraged by recent success in clinical trials. We hope that this review will stimulate further research in developing effective therapeutics, especially in developing selective RAGE inhibitors and antioxidant nanozymes that can permeate the BBB in order to treat devastating diseases such as AD.

## Data Availability

Not applicable.
